# Indications and Contra-Indications for Atropine Calabar in Eye Practice

**Published:** 1878-01

**Authors:** 


Indications and Contra-Indications for Atropine and Calabar in Eye Practice.—The *Korrespondenblatt Schweizer Aerzte* contains an address on the subject delivered by Dr. Horner, of Zurich, before the medical society of that place. He recommends the former as a narcotic (1) in pains resulting from wounds of the sub-epithelial terminal fibres of the trigeminus, also after the removal of foreign bodies, in pustules and superficial traumata of the cornea; (2) in reflex spasm, which, produced by wounds of the terminal filaments of the trigeminus, extend thence to the facial, in blepharospasm and photophobia. As a midriatic, atropine is employed for diagnostic purposes to detect changes in the bottom of the eye, the lens and the choroid; and therapeutically to provent and to cure iritis. The paralysis of accommodation produced by atropine is sometimes utilized in certain spasms of accommodation, and in the cure of certain forms of progressive myopia in children. It should never be employed in glaucoma, and must be cautiously used whenever there is a tendency to atrophic conjunctivitis, and in cases of marked injection of the conjunctiva.

Calabar, possessing a myotic action, is ordered in the form of eserin or physostigmin in cases of natural or artificial midriasis, before the iridectomy for glaucoma, in very large corneal ulcers and iris-prolapses of accommodation, and in those forms of glaucoma in which the intra-ocular tension is not sufficiently reduced after iridectomy.—*Medizinischoe Neaigkeiten*, November 17, 1877.

